# Supplementary material for: Association between integrase strand transfer inhibitor (INSTIs) use with insulin resistance and incident diabetes mellitus in persons living with HIV: A systematic review and meta-analysis protocol
Source: PLoS One. 2022 Mar 2;17(3):e0264792. doi: 10.1371/journal.pone.0264792 (PMC8890726; doi:10.1371/journal.pone.0264792)
Supplement: S1 Checklist — (DOC) [file pone.0264792.s001.doc]

Association between integrase strand transfer inhibitor (INSTIs) use with insulin resistance and incident diabetes mellitus in persons living with HIV: A systematic review and meta-analysis protocol.

**Supporting information S1;** Performance of the proposed systematic review protocol on the PRISMA-P (Preferred Reporting Items for Systematic review and Meta-Analysis Protocols) 2015 checklist.

| Section and topic | Item No | Checklist item | Is the item included in the proposed protocol? |
| --- | --- | --- | --- |
| ADMINISTRATIVE INFORMATION | | |  |
| Title: |  |  |  |
| Identification | 1a | Identify the report as a protocol of a systematic review | Yes (page 1) |
| Update | 1b | If the protocol is for an update of a previous systematic review, identify as such |  |
| Registration | 2 | If registered, provide the name of the registry (such as PROSPERO) and registration number | PROSPERO: [CRD42021273040](https://www.crd.york.ac.uk/prospero/display_record.php?ID=CRD42021273040). |
| Authors: |  |  |  |
| Contact | 3a | Provide name, institutional affiliation, e-mail address of all protocol authors; provide physical mailing address of corresponding author | Yes (page 1) |
| Contributions | 3b | Describe contributions of protocol authors and identify the guarantor of the review |  |
| Amendments | 4 | If the protocol represents an amendment of a previously completed or published protocol, identify as such and list changes; otherwise, state plan for documenting important protocol amendments | NA |
| Support: |  |  |  |
| Sources | 5a | Indicate sources of financial or other support for the review | Yes (page 10, line 227-228) |
| Sponsor | 5b | Provide name for the review funder and/or sponsor |  |
| Role of sponsor or funder | 5c | Describe roles of funder(s), sponsor(s), and/or institution(s), if any, in developing the protocol |  |
| INTRODUCTION | | |  |
| Rationale | 6 | Describe the rationale for the review in the context of what is already known | Yes (page 4, line 73-100) |
| Objectives | 7 | Provide an explicit statement of the question(s) the review will address with reference to participants, interventions, comparators, and outcomes (PICO) | Yes (page 5, line 104-108) |
| METHODS | | |  |
| Eligibility criteria | 8 | Specify the study characteristics (such as PICO, study design, setting, time frame) and report characteristics (such as years considered, language, publication status) to be used as criteria for eligibility for the review | Yes (page 6, line 117-133) |
| Information sources | 9 | Describe all intended information sources (such as electronic databases, contact with study authors, trial registers or other grey literature sources) with planned dates of coverage | Yes (page 8,line 164-174) |
| Search strategy | 10 | Present draft of search strategy to be used for at least one electronic database, including planned limits, such that it could be repeated | Yes (page 5, line 165-166) |
| Study records: |  |  | Yes (page 8, 164-182) |
| Data management | 11a | Describe the mechanism(s) that will be used to manage records and data throughout the review |  |
| Selection process | 11b | State the process that will be used for selecting studies (such as two independent reviewers) through each phase of the review (that is, screening, eligibility and inclusion in meta-analysis) | Yes |
| Data collection process | 11c | Describe planned method of extracting data from reports (such as piloting forms, done independently, in duplicate), any processes for obtaining and confirming data from investigators | Yes |
| Data items | 12 | List and define all variables for which data will be sought (such as PICO items, funding sources), any pre-planned data assumptions and simplifications | Yes |
| Outcomes and prioritization | 13 | List and define all outcomes for which data will be sought, including prioritization of main and additional outcomes, with rationale | Yes (page 7, line 150-152) |
| Risk of bias in individual studies | 14 | Describe anticipated methods for assessing risk of bias of individual studies, including whether this will be done at the outcome or study level, or both; state how this information will be used in data synthesis | Yes (page 8, line 183-190) |
| Data synthesis | 15a | Describe criteria under which study data will be quantitatively synthesised | Yes (page 9, lines 191-204) |
|  | 15b | If data are appropriate for quantitative synthesis, describe planned summary measures, methods of handling data and methods of combining data from studies, including any planned exploration of consistency (such as I^2^, Kendall’s τ) | Yes |
|  | 15c | Describe any proposed additional analyses (such as sensitivity or subgroup analyses, meta-regression) | Yes |
|  | 15d | If quantitative synthesis is not appropriate, describe the type of summary planned | NA |
| Meta-bias(es) | 16 | Specify any planned assessment of meta-bias(es) (such as publication bias across studies, selective reporting within studies) | Yes (page 8, line 183-190) |
| Confidence in cumulative evidence | 17 | Describe how the strength of the body of evidence will be assessed (such as GRADE) | Yes (page 9, line 208-216) |

*From: Shamseer L, Moher D, Clarke M, Ghersi D, Liberati A, Petticrew M, Shekelle P, Stewart L, PRISMA-P Group. Preferred reporting items for systematic review and meta-analysis protocols (PRISMA-P) 2015: elaboration and explanation. BMJ. 2015 Jan 2;349(jan02 1):g7647.*

**Supporting information S2:** Preliminary data search -Karolinska University Library search consultation group

Date: 21^st^- May 2021

Topic/research question: Association between integrase strand inhibitor (INSTIs) use with incident diabetes mellitus and/or metabolic syndrome in persons living with HIV: a systematic review and meta-analysis.

Name of researcher(s): Frank Mulindwa, Habiba Kamal & Nele Brusselaers.

Librarian(s): GunBrit Knutssön & Narcisa Hannerz

Databases:

1. Medline(OVID)
2. Embase.com
3. Web of Science(Clarivate)

Total number of hits:

- Before deduplication: 13,543
- After deduplication: 7,509

**Table 1**

1. Medline

| Interface: Ovid MEDLINE(R) and Epub Ahead of Print, In-Process & Other Non-Indexed Citations and Daily  Date of Search: 19^th^ of May 2021  Number of hits: 3,878  Comment: In Ovid, two or more words are automatically searched as phrases; i.e. no quotation marks are needed | Field labels   - exp/ = exploded MeSH term - / = non exploded MeSH term - .ti,ab,kf. = title, abstract and author keywords - adjx = within x words, regardless of order - * = truncation of word for alternate endings |
| --- | --- |
| | **#** | **Searches** | **Results** | | --- | --- | --- | | 1 | exp Anti-Retroviral Agents/ | 80301 | | 2 | Highly Active Antiretroviral Therapy/ | 21856 | | 3 | ((agent* or drug* or inhibitor* or therapy) adj1 (anti-aids or anti-hiv or anti-retroviral or antiretroviral or hiv integrase)).ti,ab,kf. | 55929 | | 4 | (bictegravir or cabotegravir or dolutegravir or elvitegravir or raltegravir or HAART).ti,ab,kf. | 15389 | | 5 | or/1-4 | 113300 | | 6 | Blood Glucose/ | 168845 | | 7 | exp Diabetes Mellitus/ | 443615 | | 8 | Glycated Hemoglobin A/ | 36624 | | 9 | Homeostasis/ | 63744 | | 10 | exp Hyperglycemia/ | 37410 | | 11 | Hyperinsulinism/ | 8671 | | 12 | exp Insulins/ | 193888 | | 13 | exp Insulin Resistance/ | 86966 | | 14 | (diabetes or insulin-dependent or non-insulin dependent or prediabetic).ti,ab,kf. | 572592 | | 15 | (blood glucose or fasting glucose or glucose intolerance or hemoglobin* or homeosta* or HOMA or hyperglyc?em* or insulin* or hyperinsulin*).ti,ab,kf. | 770564 | | 16 | ((complicat* or syndrome) adj1 (cardiometabolic or cardiovascular or dysmetabolic or metabolic or reaven)).ti,ab,kf. | 70939 | | 17 | (DM1 or DM2 or IDDM or Hb A1 or HbA1 or Hb A1c or HbA1c or MODY or NIDDM or T1D or T2D).ti,ab,kf. | 72094 | | 18 | or/6-17 | 1338765 | | 19 | 5 and 18 | 4383 | | 20 | exp HIV Infections/ | 291585 | | 21 | (acquired immunodeficiency syndrome or AIDS or HIV).ti,ab,kf. | 408858 | | 22 | or/20-21 | 449506 | | 23 | 5 and 18 and 22 | 4115 | | 24 | limit 23 to yr="2000 -Current" | 3878 | | |

2. Embase

| Interface: embase.com  Date of Search: 19^th^ of May 2021  Number of hits: 5,175  Comment: Emtree is the controlled vocabulary in Embase | Field labels   - /exp = exploded Emtree term - /de = non exploded Emtree term - ti,ab,kw = title, abstract and author keywords - NEAR/x = within x words, regardless of order - * = truncation of word for alternate endings |
| --- | --- |
| #1 'antiretrovirus agent'/exp/mj #2 'highly active antiretroviral therapy'/mj  #3 ((agent* OR drug* OR inhibitor* OR therapy) NEAR/1 ('anti aids' OR 'anti hiv' OR 'anti retroviral' OR antiretroviral OR 'hiv integrase')):ti,ab,kw #4 bictegravir:ti,ab,kw OR cabotegravir:ti,ab,kw OR dolutegravir:ti,ab,kw OR elvitegravir:ti,ab,kw OR raltegravir:ti,ab,kw OR haart:ti,ab,kw #5 #1 OR #2 OR #3 OR #4  #6 'glucose blood level'/mj #7 'diabetes mellitus'/exp/mj #8 'glycosylated hemoglobin'/exp/mj #9 'homeostasis'/mj #10 'hyperglycemia'/mj #11 'glucose intolerance'/mj #12 'hyperinsulinism'/mj #13 'insulin derivative'/exp/mj #14 'insulin resistance'/mj #15 'metabolic syndrome x'/mj #16 diabetes:ti,ab,kw OR 'insulin-dependent':ti,ab,kw OR 'non-insulin dependent':ti,ab,kw OR prediabetic:ti,ab,kw #17 'blood glucose':ti,ab,kw OR 'fasting glucose':ti,ab,kw OR 'glucose intolerance':ti,ab,kw OR hemoglobin*:ti,ab,kw OR homeosta*:ti,ab,kw OR homa:ti,ab,kw OR hyperglyc$em*:ti,ab,kw OR insulin*:ti,ab,kw OR hyperinsulin*:ti,ab,kw #18 ((complicat* OR syndrome) NEAR/1 (cardiometabolic OR cardiovascular OR dysmetabolic OR metabolic OR reaven)):ti,ab,kw #19 'dm1':ti,ab,kw OR 'dm2':ti,ab,kw OR 'iddm':ti,ab,kw OR 'hb a1':ti,ab,kw OR 'hba1':ti,ab,kw OR 'hb a1c':ti,ab,kw OR 'hba1c':ti,ab,kw OR 'mody':ti,ab,kw OR 'niddm':ti,ab,kw OR 't1d':ti,ab,kw OR 't2d':ti,ab,kw #20 #6 OR #7 OR #8 OR #9 OR #10 OR #11 OR #12 OR #13 OR #14 OR #15 OR #16 OR #17 OR #18 OR #19  #21 #5 AND #20  #22 'human immunodeficiency virus infection'/exp/mj #23 'acquired immunodeficiency syndrome':ti,ab,kw OR aids:ti,ab,kw OR hiv:ti,ab,kw OR "human immunodeficiency virus $ infection':ti,ab,kw  #24 #22 OR #23  #25 #21 AND #24 AND [2000-2021]/py | |

3. Web of Science Core Collection

| Interface: Clarivate Analytics  Date of Search: 19^th^ of May 2021  Number of hits: 4,490 | Field labels   - TS/Topic = title, abstract, author keywords and Keywords Plus - NEAR/x = within x words, regardless of order - * = truncation of word for alternate endings   Note: sometimes “quotation marks” are needed for single search terms to avoid automatic term mapping (lemmatization). |
| --- | --- |
| #1 ((agent* or drug* or inhibitor* or therapy) NEAR/1 (“anti-aids” or “anti-hiv” or anti-retroviral or antiretroviral or “hiv integrase”)) OR (bictegravir or cabotegravir or dolutegravir or elvitegravir or raltegravir or HAART)  #2 (diabetes or “insulin-dependent” or “non-insulin dependent” or prediabetic) OR  (“blood glucose” or “fasting glucose” or “glucose intolerance” or hemoglobin* or homeosta* or HOMA or hyperglyc$em* or insulin* or hyperinsulin*) OR ((complicat* or syndrome) NEAR/1 (cardiometabolic or cardiovascular or dysmetabolic or metabolic or reaven)) OR  (DM1 or DM2 or IDDM or “Hb A1” or HbA1 or “Hb A1c” or HbA1c or MODY or NIDDM or T1D or T2D)  #3 (“acquired immunodeficiency syndrome” or AIDS or HIV)  #4 #1 AND #2 AND #3 Refined by: PUBLICATION YEARS: 2000- 2021 | |
