# Supplementary material for: Association between integrase strand transfer inhibitor (INSTIs) use with insulin resistance and incident diabetes mellitus in persons living with HIV: A systematic review and meta-analysis protocol
Source: PLoS One. 2022 Mar 2;17(3):e0264792. doi: 10.1371/journal.pone.0264792 (PMC8890726; doi:10.1371/journal.pone.0264792)
Supplement: Attachment — Submitted filename: Response to reviewers.docx [file pone.0264792.s002.docx]

20^th^ – January – 2022

To the academic editor

The PLOS ONE journal

Dear Editor.

We wish to express our gratitude for the opportunity to submit a revised version of the manuscript after the valuable feedback from you and the reviewers. We have now addressed all comments, and we have incorporated the suggestions. Please find all our replies point by point below in this rebuttal letter and all the changes highlighted in the revised manuscript.

Re: Response to comments raised by reviewers on review of our manuscript titled; **Association between integrase strand transfer inhibitor (INSTIs) use with insulin resistance and incident diabetes mellitus in persons living with HIV: A systematic review and meta-analysis protocol**.

>> Reply: We were pleased that our manuscript was found worthy of a full review by the PLOS editorial team. We are committed to making it even better hence we have taken into effect all the comments that were raised as shown below:

**General comments from the editorial team:**

*Please ensure that your manuscript meets PLOS ONE's style requirements, including those for file naming. The PLOS ONE style templates can be found at*

[*https://journals.plos.org/plosone/s/file?id=wjVg/PLOSOne_formatting_sample_main_body.pdf*](https://journals.plos.org/plosone/s/file?id=wjVg/PLOSOne_formatting_sample_main_body.pdf)  *and* [*https://journals.plos.org/plosone/s/file?id=ba62/PLOSOne_formatting_sample_title_authors_affiliations.pdf*](https://journals.plos.org/plosone/s/file?id=ba62/PLOSOne_formatting_sample_title_authors_affiliations.pdf)

>> Reply: The whole manuscript has been edited to take into effect all the highlights of PLOS ONE’s style requirements as directed by the attached links.

*2. In your Data Availability statement, you have not specified where the minimal data set underlying the results described in your manuscript can be found. PLOS defines a study's minimal data set as the underlying data used to reach the conclusions drawn in the manuscript and any additional data required to replicate the reported study findings in their entirety. All PLOS journals require that the minimal data set be made fully available. For more information about our data policy, please see* [*http://journals.plos.org/plosone/s/data-availability*](http://journals.plos.org/plosone/s/data-availability)*.*

>> Reply: The data availability statement was initially not put in the manuscript. This has been introduced in lines 205-207, page 9 highlighting that the dataset analyzed will be attached in the meta-analysis manuscript as supplementary material. For the current manuscript, there is no data to attach since it is a protocol.

*Upon re-submitting your revised manuscript, please upload your study’s minimal underlying data set as either Supporting Information files or to a stable, public repository and include the relevant URLs, DOIs, or accession numbers within your revised cover letter. For a list of acceptable repositories, please see* [*http://journals.plos.org/plosone/s/data-availability#loc-recommended-repositories*](http://journals.plos.org/plosone/s/data-availability#loc-recommended-repositories)*. Any potentially identifying patient information must be fully anonymized.*

*Important: If there are ethical or legal restrictions to sharing your data publicly, please explain these restrictions in detail. Please see our guidelines for more information on what we consider unacceptable restrictions to publicly sharing data:* [*http://journals.plos.org/plosone/s/data-availability#loc-unacceptable-data-access-restrictions*](http://journals.plos.org/plosone/s/data-availability#loc-unacceptable-data-access-restrictions)*. Note that it is not acceptable for the authors to be the sole named individuals responsible for ensuring data access.*

*We will update your Data Availability statement to reflect the information you provide in your cover letter.*

>> Reply: This does not apply to the current manuscript because it is a protocol manuscript

*3. We note you have included a table to which you do not refer in the text of your manuscript. Please ensure that you refer to Table 1 in your text; if accepted, production will need this reference to link the reader to the Table.*

>> Reply: This has been rectified in lines;134-141, page 6, **study outcomes**

*4. Please review your reference list to ensure that it is complete and correct. If you have cited papers that have been retracted, please include the rationale for doing so in the manuscript text, or remove these references and replace them with relevant current references. Any changes to the reference list should be mentioned in the rebuttal letter that accompanies your revised manuscript. If you need to cite a retracted article, indicate the article’s retracted status in the References list and also include a citation and full reference for the retraction notice.*

>> Reply: The references have been reviewed

**Reviewers’ comments:**

*Reviewer #1: This is an important study and the manuscript reads well. Overall, it is technically sound. I did have a number of queries and considerations which may be pertinent.

1. Could the authors explain how they will account for prior ART exposure, and previous ART regimens*

>> Reply: We plan to perform a sub -group analysis to evaluate the effect on integrase inhibitors on incident diabetes and insulin resistance in ART naïve patients and ART exposed patients. This is highlighted on page 9 (data synthesis, lines; 196-201; Forest plots will be created to visually inspect and analyse the effect sizes and corresponding 95% CI. If possible, subgroup analysis or meta-regression (if more or equal to 10 studies) will be performed for crude and adjusted estimates, particular integrase inhibitor, study designs, sex, age, duration of follow-up, geographical origin of the study population, **ART status on enrolment into the study (first- or second-line treatment)**, and other HIV-related factors to assess contribution to effect sizes.

However, we agree with the reviewer that certain types of ART (previous exposure) may confound the present risk to diabetes more than other types. We have included this in the summary page- page 2- limitations

*2. The period of study is over a large time period January 2000 to May 2021. There have been several clinical management developments during this time which may have a bearing. Have the authors considered this and might a shorter time period of review be more advantageous in this respect.*

>> Reply: We used that timeline so as to capture phase III trials comparing efficacy of integrase inhibitors as compared to protease inhibitors and non-nucleoside reverse transcriptase inhibitors. The first integrase inhibitor, Raltegravir was FDA approved in 2007 and how far backwards we searched was informed by a preliminary data search. We agree with the reviewer that over that time, various advances have been made with integrase inhibitors including the introduction of dolutegravir, elvitegravir, bictegravir and carbotegravir. We hope to account for this by performing sub group analysis according to the individual integrase inhibitor as highlighted on page 9 (data synthesis, lines; 196-201). This would help delineate whether newer generation drugs in that class are different from the older drugs in terms of glucose metabolism.

Additionally to capture new studies which would have been published, we have extended our search timeline from May 2021 to 31^st^ – January – 2022 as highlighted on page 3, line 57-62 and page 5, lines 118-120.
 *3. It is difficult to define a priori sub group analysis but some ideas of potential areas may enhance the protocol. Amongst this geographical and ethnic subgroup might provide useful insights.*

>> Reply: we added these to the protocol as follows (page 9, line 197-201); If possible, subgroup analysis or meta-regression (if more or equal to 10 studies) will be performed for crude and adjusted estimates, particular integrase inhibitor, study designs, sex, age, duration of follow-up, **geographical origin of the study population**, ART status on enrolment into the study (first- or second-line treatment), and other HIV-related factors to assess contribution to effect sizes.

*Reviewer #2: It's an interesting study. There are no studies evaluating the association between Integrase inhibitor and diabetes or insulin resistance.

The authors should use official abbreviation for drug group name.*

>> Reply: Thank you very much for the comment. We found the two acceptable abbreviations for HIV integrase inhibitors are: INI- integrase inhibitors and INSTI- integrase strand transfer inhibitors. In our manuscript we used ‘INSTI’.

Thank you very much.

Sincerely,

Dr. Frank Mulindwa,

Corresponding author.
